# On Splitting Training and Validation Set: A Comparative Study of Cross-Validation, Bootstrap and Systematic Sampling for Estimating the Generalization Performance of Supervised Learning

**DOI:** 10.1007/s41664-018-0068-2

**Published:** 2018-10-29

**Authors:** Yun Xu, Royston Goodacre

**Affiliations:** 1 School of Chemistry, Manchester Institute of Biotechnology, The University of Manchester, Manchester, M1 7DN UK; 2 Department of Biochemistry, Institute of Integrative Biology, University of Liverpool, Biosciences Building, Crown Street, Liverpool, L69 7ZB UK

**Keywords:** Cross-validation, Bootstrapping, Bootstrapped Latin partition, Kennard-Stone algorithm, SPXY, Model selection, Model validation, Partial least squares for discriminant analysis, Support vector machines

## Abstract

Model validation is the most important part of building a supervised model. For building a model with good generalization performance one must have a sensible data splitting strategy, and this is crucial for model validation. In this study, we conducted a comparative study on various reported data splitting methods. The MixSim model was employed to generate nine simulated datasets with different probabilities of mis-classification and variable sample sizes. Then partial least squares for discriminant analysis and support vector machines for classification were applied to these datasets. Data splitting methods tested included variants of cross-validation, bootstrapping, bootstrapped Latin partition, Kennard-Stone algorithm (K-S) and sample set partitioning based on joint *X*–*Y* distances algorithm (SPXY). These methods were employed to split the data into training and validation sets. The estimated generalization performances from the validation sets were then compared with the ones obtained from the blind test sets which were generated from the same distribution but were unseen by the training/validation procedure used in model construction. The results showed that the size of the data is the deciding factor for the qualities of the generalization performance estimated from the validation set. We found that there was a significant gap between the performance estimated from the validation set and the one from the test set for the all the data splitting methods employed on small datasets. Such disparity decreased when more samples were available for training/validation, and this is because the models were then moving towards approximations of the central limit theory for the simulated datasets used. We also found that having too many or too few samples in the training set had a negative effect on the estimated model performance, suggesting that it is necessary to have a good balance between the sizes of training set and validation set to have a reliable estimation of model performance. We also found that systematic sampling method such as K-S and SPXY generally had very poor estimation of the model performance, most likely due to the fact that they are designed to take the most representative samples first and thus left a rather poorly representative sample set for model performance estimation.

**Electronic supplementary material:**

The online version of this article (10.1007/s41664-018-0068-2) contains supplementary material, which is available to authorized users.

## Introduction

**Fig. 1:**
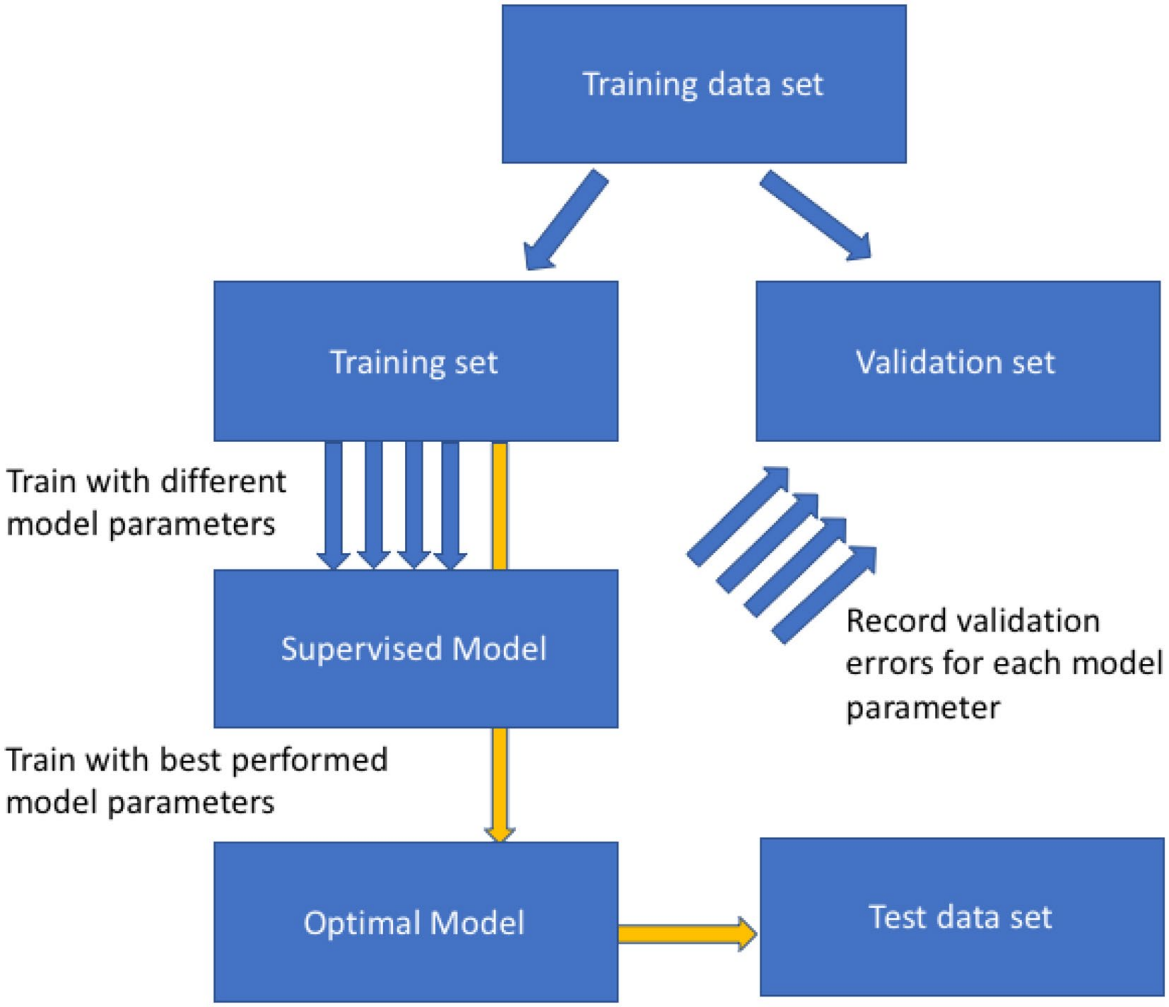
General flowchart used for model selection. Blue arrows indicate the validation process while yellow arrows indicate the final training and test on blind test set process

Supervised learning which is used for sample classification from (bio)chemical data is a very common task in chemometrics studies. Most classification models have one or more model parameters that are used to control the complexity of the model. The higher the complexity in the model the more discriminating power the model possesses, although the risk of over-fitting also increases. Over-fitting is a phenomenon often seen when a trained model performs extremely well on the samples used for training but performs poorly on new unknown samples; that is to say the model does not generalize well. To find an optimal set of model parameter(s), which have an appropriate balance between these two aspects, it is necessary to split the data into training and validation set. The training set is used to build the model with multiple model parameter settings and then each trained model is challenged with the validation set. The validation set contains samples with known provenance, but these classifications are not known to model, therefore, predictions on the validation set allow the operator to assess model accuracy. Based on the errors on the validation set, the optimal model parameter(s) set is determined using the one with the lowest validation error. This procedure is called model selection [1]. It is important to have a good estimation of the performance of the trained and optimized model on unknown samples in general, i.e., to assess the generalization performance. A couple of decades ago, it was a commonly accepted assumption that the measured performance of the model using the validation set was an unbiased estimator of the performance of such models in general. However, multiple recent studies have demonstrated that this assumption does not always hold. As demonstrated by Westerhuis et al. [2], the performance measured by cross-validation is an over-optimistic one. Harrington et al. [3] also demonstrated that a single split of training and test set can provide erroneous estimation of model performance. These studies highlight the importance in having an additional blind test set which is not used during the model selection and validation process to have a better estimation of the generalization performance of the model. A general flowchart of a typical model validation process is given in Fig. 1. However, even following this procedure (Fig. 1) it is still impossible to tell how well the estimated predictive performance of the model from the blind test set matches the true underlying distribution of the data. This is because in real-world applications the latter is normally unknown, and one has to assume that the measured performance using blind test set is an unbiased, accurate estimator for the model performance on all unknown samples coming from the same distribution of the training/test dataset. Clearly without sampling the whole of a population this is unlikely, but one assumes that with resampling one can approximate the central limit theory for that population. In addition, the estimated performance of the model is likely to be affected by many factors such as the modelling algorithm, the overlap between the data, the number of samples available for training and perhaps most importantly the method used for splitting the data.

There are many data splitting methods reported and used in the literature. These methods can be roughly categorized into three different types:

1. cross-validation (CV) [4];
2. randomly selecting a proportion of samples and retaining these (holding out) as a validation set and then using the remaining samples for training. This process is usually repeated many times and the final estimation of the model performance is the average performance on validation sets of all the repeats; of course, one can also look at IQR of the predictive powers as well. In our opinion, the best-known method used for this type of repartitioning of the data is probably the bootstrap as proposed by Efron et al. [5].
3. Based on the distribution of the data, systematically selecting a given number of the most representative samples from the datasets and using the remaining samples for validation is a third approach. Kennard-Stone algorithm (K-S) [6] is a good example of such a method.

These data splitting methods have one or two parameters that need to be optimized; e.g., the number of folds in CV, the number of iterations in the bootstrap, etc. All these methods have been routinely reported in the literature and despite their popularity, most people chose a method with which they have familiarity. Daszykowski et al. [7] presented an excellent review of data splitting methods based systematic sample selection; Puzyn et al. [8] conducted a comparative study into the effect of K-S, two of K-S variants and two closely related algorithms in QSAR studies. However, to the best of our knowledge a comprehensive comparison of the methods across all three categories, particularly with respect to the effect of choosing different parameter setting on each method, is still lacking. Therefore, in this study, we conducted a comprehensive comparative study of multiple data splitting methods commonly reported in the literature and we explored a wide range of different parameter settings. These methods include leave-one-out (LOO) CV, *k*-fold CV, Monte-Carlo CV [9], bootstrapping [5], bootstrapped Latin partition (BLP) [10], K-S and sample set partitioning based on joint *X*–*Y* distances (SPXY) [11].

The datasets we employed were simulated datasets generated by the MixSim model developed by Melnykov et al. [12]. The main advantage of this model is that it can generate true multivariate datasets (i.e., not pseudo multivariate by stacking multiple simulated discriminative variables together) with known probability of misclassification. This model was further improved by Riani et al. [13] allowing more controls on the probability of misclassification and it was incorporated to flexible statistics and data analysis (FSDA) toolbox for MATLAB. MixSim provides an excellent testing ground for examining classification algorithms and for us this also includes the various different data splitting methods. In this study, we employed the improved MixSim model implemented in FSDA to generate three underlying distributions with different known probabilities of misclassification. For each distribution, three datasets were generated containing different number of samples: 30, 100 and 1000. We then employed two commonly used multivariate classification models on these datasets including partial least squares for discriminant analysis (PLS-DA), as this is a very popular algorithm [14, 15], and support vector machines for classification (SVC) [16, 17] as the kernel needs optimization and can be used for non-linear, as well as linear classification mapping. The model training/validation was performed using the data splitting methods as listed above with a wide range of parameter settings (vide infra). The estimated model performances on the validation sets were then compared with the ones obtained from the corresponding blind test sets which were 1000 additional samples generated in MixSim from the same distribution but unknown to the training/validation procedure.

## Chemometric Methods

In this section, a brief description of the MixSim model is given, followed by a short review of all the data splitting methods used in this study. Since the descriptions for PLS-DA and SVC had already been extensively reported in the literature [14–17] they will not be repeated here.

### MixSim Model

The MixSim model is essentially a multivariate finite mixed normal distribution of *c* classes in *v* dimension. Each class is defined by a covariance matrix ***C*** and a mean vector $\varvec{\mu}$. The probability of misclassification (i.e., overlap) between class *i* and *j*, denoted as $\omega_{j|i}$, is formulated as the cumulative distribution function of linear combinations of *v* independent non-central *χ*^2^ random variables and *v* normal random variables. The probability of misclassification $\omega_{j|i}$ can be calculated using Eq. (1):

1 $$\omega_{j|i} = { \Pr }_{{N_{p} (\mu_{i} , C_{i} )}} \left[ {\mathop \sum \limits_{{\begin{array}{*{20}c} {l = 1} \\ {l:\lambda_{l} \ne 1} \\ \end{array} }}^{v} \left( {\lambda_{l} - 1} \right)U_{l} + 2\mathop \sum \limits_{{\begin{array}{*{20}c} {l = 1} \\ {l:\lambda_{l} \ne 1} \\ \end{array} }}^{v} \delta_{l} W_{l} \le \mathop \sum \limits_{{\begin{array}{*{20}c} {l = 1} \\ {l:\lambda_{l} \ne 1} \\ \end{array} }}^{v} \frac{{\lambda_{l} \delta_{l}^{2} }}{{\lambda_{l} - 1}} - \mathop \sum \limits_{{\begin{array}{*{20}c} {l = 1} \\ {l:\lambda_{l} = 1} \\ \end{array} }}^{v} \delta_{l}^{2} + { \log }\left( {\frac{{\pi_{j}^{2} \left| {\varvec{C}_{\varvec{i}} } \right|}}{{\pi_{i}^{2} \left| {\varvec{C}_{\varvec{j}} } \right|}}} \right)} \right],$$

where $\delta_{l} = \gamma_{l}^{'} \varvec{C}_{i}^{{ - \frac{1}{2}}} \left( {\varvec{\mu}_{i} -\varvec{\mu}_{j} } \right)$, $\pi_{i}$ and $\pi_{j}$ are the probabilities of occurrences of class *i* and *j,* respectively; $\lambda_{l}$ and $\varvec{\gamma}_{l}$ are the eigenvalues and eigenvectors of $\varvec{C}_{j|i} = \varvec{C}_{i}^{1/2} \varvec{C}_{j}^{ - 1} \varvec{C}_{i}^{1/2}$; *U*_*l*_ is a collection of independent noncentral *χ*^2^ random variables with one degree of freedom and noncentrality parameter of $\lambda_{l}^{2} \delta_{l}^{2} /\left( {\lambda_{l} - 1} \right)^{2}$, *W*_*l*_ is a collection of independent *N*(0,1) random variables.

MixSim generates simulated data by first determining the parameters of a mixed normal distribution which would match the specified overlap. This is achieved in four steps:

1. Specify the number of classes *k*, number of variables *v*. The desired overlap $\omega$ is determined by setting two out of three parameters: the mean overlap $\bar{\omega }$, the maximum overlap $\omega_{ \hbox{max} }$ and the standard deviation of overlap $\sigma_{\omega }$. One can also determine the size of the samples in each class by giving the probability of occurrence of each class $\pi_{1} ,\varvec{ }\pi_{2} , \ldots ,\varvec{ }\pi_{c} ,\varvec{ }$ subject to $\mathop \sum \nolimits_{l = 1}^{c} \pi_{c} = 1$. The sample size of each class is then drawn from a multinomial distribution with such occurrence probabilities.
2. Generate mean vectors independently and uniformly from a *v*-variate hypercube. Random covariance matrices are initially drawn from a Wishart distribution. This step is repeated if these parameters bring to an asymptotic $\bar{\omega }$ (or $\omega_{ \hbox{max} }$) larger than the desired $\bar{\omega }$ (or $\omega_{ \hbox{max} }$).
3. Estimate the pairwise probabilities using Eq. (1) and calculate resulting $\bar{\omega }$.
4. If the calculated $\bar{\omega }$ or $\omega_{max}$ are close enough to the specified targets, the algorithm stops; otherwise, the covariance matrices were inflated or deflated by multiplying each ***C*** with a positive scaling factor and return to step (3).

After the parameters are determined, a given number of samples can be generated from the mixed normal distribution and labels were assigned to these samples accordingly.

### Data Splitting Methods

#### Cross-Validation (CV)

CV is probably the most commonly used data splitting method in model selection. It divides the data into *k* different parts (referred to as *k*-folds). One part (fold) is held out as the validation set. The model is trained on the remaining *k*-1 parts (or folds) and then applied to the validation set and record its predictive performance. This process repeated *k* times so that each part has been used as a validation set once. The recorded predictive performances are then averaged, the optimal model parameter is determined as the one that had the best averaged predictive performance. This method is often under the name of *k*-fold CV and a special case when *k* = *n* (i.e., where *n* = the total number of samples) is called leave-one-out cross-validation (LOO-CV). As one of the oldest data splitting method, there are abundant applications of CV reported in literatures.

#### Bootstrap and Monte-Carlo Cross-Validation (MCCV)

The bootstrap is a data resampling method for estimating the statistical parameters of an unknown distribution such as mean, median, variance, confidence interval, etc. [18]. It has been later proved to be a good resampling method for model selection [19]. Given *n* samples available in the data, bootstrap randomly chose *n* samples with replacement; i.e., the same sample can be chosen multiple times. These samples are used as the training set and the unselected samples are used as the validation set. The ratio of the samples in training and validation set is variable and on average 63.2% samples would be used as a training set and 36.8% samples would be used as a validation set. This process is repeated *t* times (e.g., *t* = 100) and the predictive performance of the validation sets of those repeats are recorded and averaged as the final estimation of the generalization performance of the model.

Although MCCV [9] includes the term CV (viz. cross-validation) it shares more similarity with bootstrap than *k*-fold CV or LOO-CV. Like bootstrap, MCCV randomly chose a subset of samples and used as training set to train the model and the unselected samples are used as a validation set to calculate the predictive performance of the trained model. This process is also to be repeated *t* times and the final estimated performance is the averaged predictive performance of the validation sets of these repeats. The difference in MCCV is that the random sampling is conducted without replacement, instead one needs to specify the number of samples to be used for training (*n*_*t*_).

#### Bootstrapped Latin Partition (BLP)

**Fig. 2:**
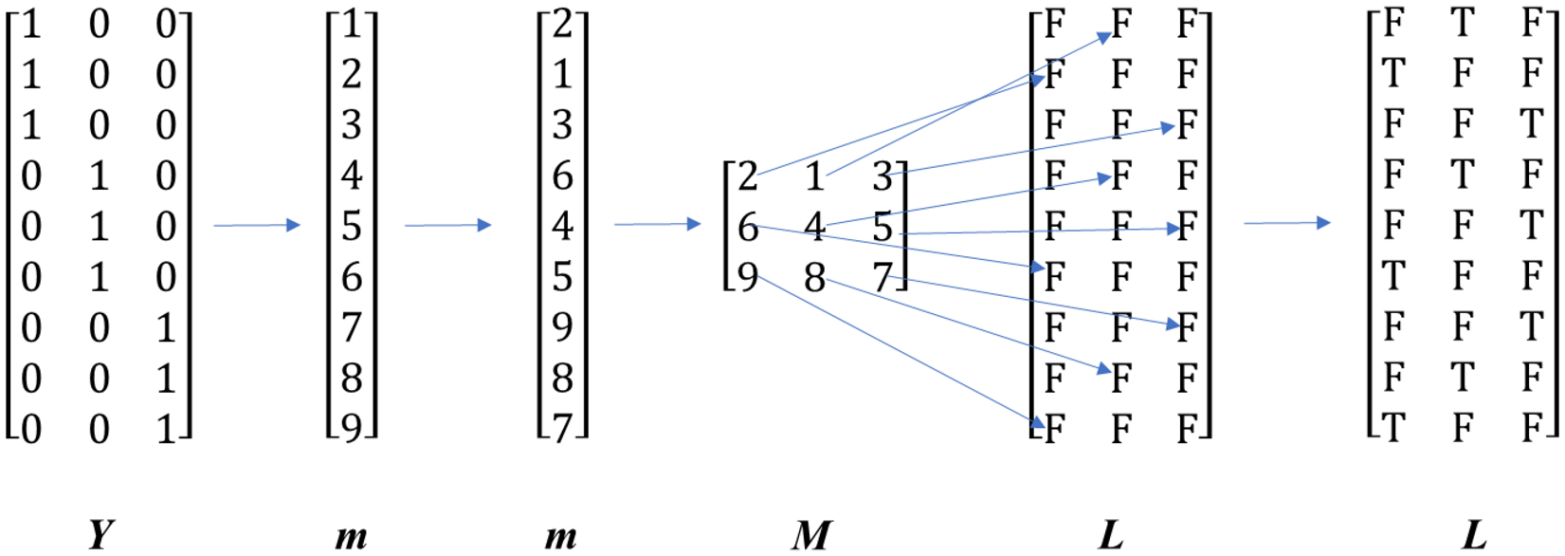
A schematic of the BLP algorithm. ***Y*** is binary coded class membership matrix, ***m*** is index vector, ***M*** is reshaped index matrix and ***L*** is a logical matrix of specifying which samples shall be used for validation in which *F* is logical *false* and *T* is logical *true*

BLP [8] can be considered as a within-class permuted *k*-fold CV. In BLP, the number of partitions (i.e., splits) *k* is to be specified by the user. For *m* partitions, *m* mutually exclusive data splitting indices are generated and ~ 1/*m* of the samples are used for validation, and the remaining samples are used for training. Then the row indices for a class are selected, randomized and concatenated to form a long vector ***k*** containing the indices of all classes. This vector is then reshaped to a *n*/*k* × *k* matrix ***K*** with the indices in ***k*** filling ***K*** along row direction. Another all *false* logic matrix ***L*** is created with the same size as ***K***. An index in ***K*** in column *a* defines the element in the corresponding row of ***L*** in column *a* to be set to be *true*. As a result, each column in ***L*** defines a split of training and validation sets, the *true* elements are the ones to be used for validation and the ones with false are to be used for training. This method is best illustrated using a real number example as shown in Fig. 2.

BLP has combined merits of random selection methods and systematic cross-validation. The class distribution is well preserved on both training and test set while all samples are used for testing, for only once. Also, due to its random nature, upper and lower bound of model performance can also be estimated through repeating the process multiple times [3].

#### Kennard-Stone algorithm (K-S) and Sample Set Partitioning Based on Joint *X*–*Y* Distances Algorithm (SPXY)

The K-S algorithm [6], also known as computer-aided design of experiment (CADEX) algorithm, is designed to select most representative samples from a given dataset. K-S employed a stepwise procedure. In the first step, the Euclidean distance between each pair of samples was calculated between each pair of samples and a pair of samples with the largest distance was chosen and ranked as most representative. Then in each following step, the remaining samples having the greatest distance from the already selected samples is chosen and added to the bottom of the previous rank list. This procedure is repeated until a pre-defined number of samples had been chosen and ranked. These selected samples are usually used as the training set since a representative dataset is crucial for training a good model and the remaining samples are used as validation set. Unlike CV and bootstrap, there is only one split of training and validation set in K-S algorithm.

SPXY [11] algorithm is based on the same idea of K-S algorithm, the only difference is that SPXY uses a composite distance as shown in Eq. (2) which measures the distance in both data matrix ***X*** and the target vector/matrix ***Y***.

2 $$d_{xy} \left( {p,q} \right) = \frac{{d_{x} (p,q)}}{{{ \hbox{max} }_{p,q \in [1,n]} d_{x} \left( {p,q} \right)}} + \frac{{d_{y} (p,q)}}{{{ \hbox{max} }_{p,q \in [1,n]} d_{y} \left( {p,q} \right)}},$$

where $$d_{x} (p,q) = ||x_{p} - x_{q}||$$, $$d_{y} (p,q) = ||y_{p} - y_{q}||$$ and $$p,q \in [1,n]$$.

The rest of the sample partitioning is as in the K-S algorithm.

## Experiment Design and Software

In this study, three different ten-dimensional (i.e., the number of input variables was set to 10) mixed normal distributions, denoted as *p*1, *p*2 and *p*3, were generated using the MixSim model as described above. The expected probabilities of misclassification are listed in Table 1. Based on these probabilities, the expected correct classification rates of a “perfect” classification model applied to the data drawn from these populations were 97.5, 90 and 65.6% for *p*1, *p*2 and *p*3, respectively. These three distributions represent three different classification problems:

**Table 1:** A confusion matrix depicting the probability of misclassification in the three distributions used to generate simulated dataset (*p*1, *p2* and *p*3)

Distribution		Class 1	Class 2	Class 3
*p*1	Class 1	–	0.0053	0.0279
	Class 2	0.0054	–	0.0049
	Class 3	0.0259	0.0056	–
*p*2	Class 1	–	0.1394	0.0177
	Class 2	0.1106	–	0.0142
	Class 3	0.0094	0.0086	–
*p*3	Class 1	–	0.1611	0.1363
	Class 2	0.1884	–	0.1317
	Class 3	0.2137	0.1998	–

1. an easy problem with mild overlap (*ω* < 3%) between each pair of classes;
2. one class is well separated from the other two (*ω* < 2%) and the remaining two classes have significant overlap between each other (*ω* > 10%); and
3. a difficult classification problem with 13–21% overlap between each pair of classes.

For each distribution, three simulated datasets are generated with 30, 100 and 1000 samples, respectively, and thus a total number of nine simulated datasets were generated for model training and validation. Finally, an additional dataset with 1000 samples was generated for each population (*p*1, *p*2 and *p*3) and they were used as blind test set. We denote the collection of datasets generated from *p*1, *p*2 and *p*3 as data1, data2 and data3, respectively. It is worth noting that in real applications, training, validation and test set are drawn from the same dataset. The reason we chose to have an external test set with a large number of samples is that this enables a stable estimation of model performance, not to be affected by other factors such as sample size, data splitting methods. This is vital for a fair comparison across different combinations of datasets, data splitting methods and their parameter settings. Of course, this is only possible with simulated data with access to unlimited samples. In real applications, we highly recommend users to repeat the model validation process multiple times with different combinations of all three sets to assess the stability of the estimation of the model performance.

PLS-DA and SVC with linear kernel, which are popular for classification [20], were used as classification models and applied to the nine simulated datasets. Both models have a single model parameter which need to be optimized: the number of PLS components for PLS-DA and the cost parameter for SVC. For PLS-DA, the number of PLS components was varied from 1 to 10 and for SVC the cost parameter was varied from 2^−14^ to 2^14^ in log2 space. We intentionally set a wide choice of model parameter candidates so that the optimal parameter will be missed because it was not included. This was done to test whether, or how often, the validation process can be drawn to some unreasonable parameter settings. The PLS-DA class membership assignment was determined by assigning the test sample to the class with largest predicted output while one-vs-one approach [16] was employed for SVC class membership prediction.

LOO-CV, *k*-fold CV, BLP, bootstrap, MCCV, K-S and SPXY with a wide range of parameter settings were applied to split each dataset into training and validation set and used to train the models and find optimal model parameters. The parameter settings of these methods that we used are listed below:

1. *k*-fold CV: *k* was set to be 3, 7 and 10.
2. BLP: *k* was set to be 2, 3, … 10. We followed Harrington’s recommendation and each BLP splitting was repeated four times and the averaged results were reported.
3. Bootstrap: *t* was set to be 10, 100 and 1000.
4. MCCV: *n*_*t*_ was set to be 10, 20, … 90% of the dataset and *t* was set to be 10, 100 and 1000, every combination of *n*_*t*_ and *t* was tested.
5. K-S: 10, 20, … 80% of top-ranked samples in the dataset was selected as the training set.
6. SPXY: used the same parameters as K-S.

Again, it was intentional to test a wide range of parameters, this was to demonstrate the effect of using some unreasonable parameter settings on model selection; e.g., K-S with the 10% top-ranked samples to be used for training when this was applied to a dataset with 30 samples, the training set would only contain three samples. Once the optimal model parameters were decided, the model was trained again on the full data with training and validation set combined using the optimal model parameter and applied to the blind test set to assess its generalization performance.

All the calculations were conducted on MATLAB 2017a (Mathworks, MA, US.). FSDA toolbox for MATLAB was obtained from the Ro.S.A. website at [21]; BLP was implemented as a MATLAB function using the code provided in the supporting information of Ref. [10]; SPXY was implemented as a MATLAB function using the code provide in the supporting information of Ref. [11]; SVC was implemented using LibSVM toolbox [22]. Liblinear [23], a variant of LibSVM toolbox was used for analyzing datasets with 1000 samples as it has much faster training speed on large datasets; PLS-DA was performed using *plsregress* function in the MATLAB Statistics toolbox, all other calculation was performed using in-house MATLAB scripts which are freely available on our GitHub repository at https://github.com/biospec.

## Results and Discussion

**Fig. 3:**
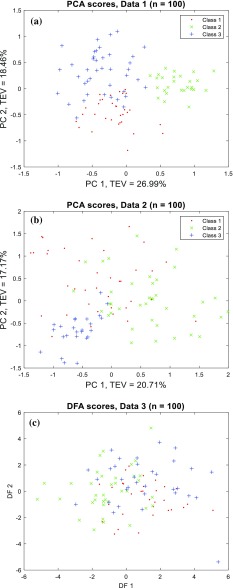
PCA scores plot of **a** data1 (*p*1); and **b** of data2 (*p*2); and **c** a DFA scores plot of data3 (*p*3). All scores plots are constructed with 100 samples in each of the datasets

To give an intuitive view of the patterns within the datasets drawn of the three distributions as described above we conducted principal component analysis (PCA) [24]. The PCA scores plots of principal component 1 (PC1) *versus* PC2 for data1 and data2, each containing 100 samples, are shown in Fig. 3a, b and one can see the different classes and the overlap between them. The overlap in data3 was too high, and therefore, PCA was unable to show any separations between classes in first 3 PCs (data not shown), a scores plot of discriminant function analysis (DFA) [25] applied directly to data3 with 100 samples was provided instead in Fig. 3c.

**Fig. 4:**
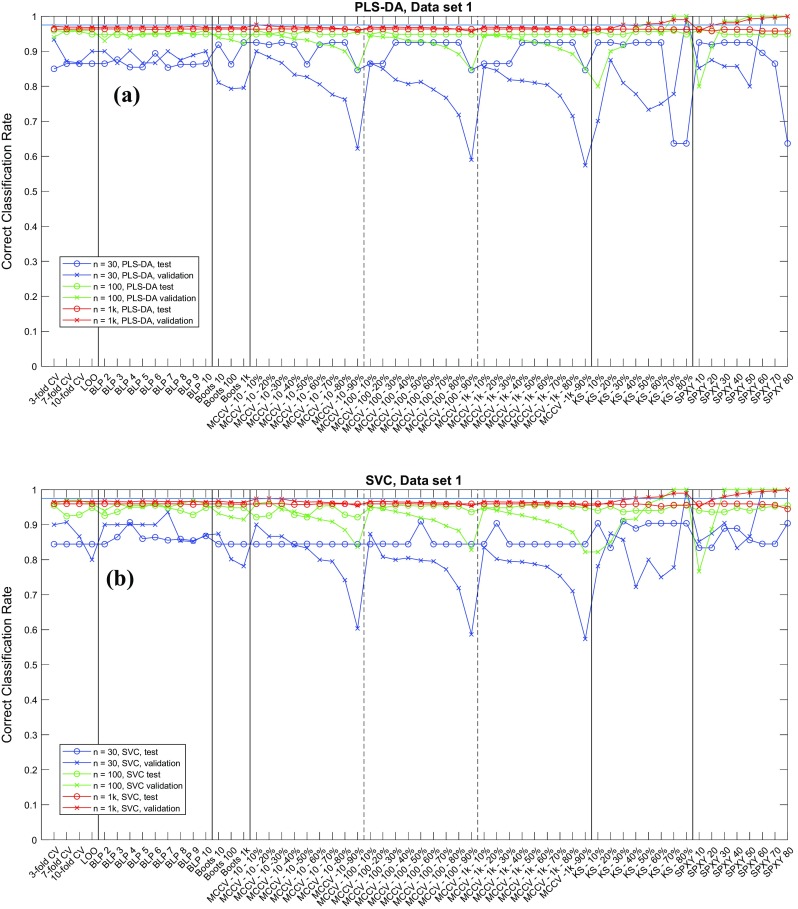
CCRs on data1 (*p*1) for **a** PLS-DA and **b** SVC

**Fig. 5:**
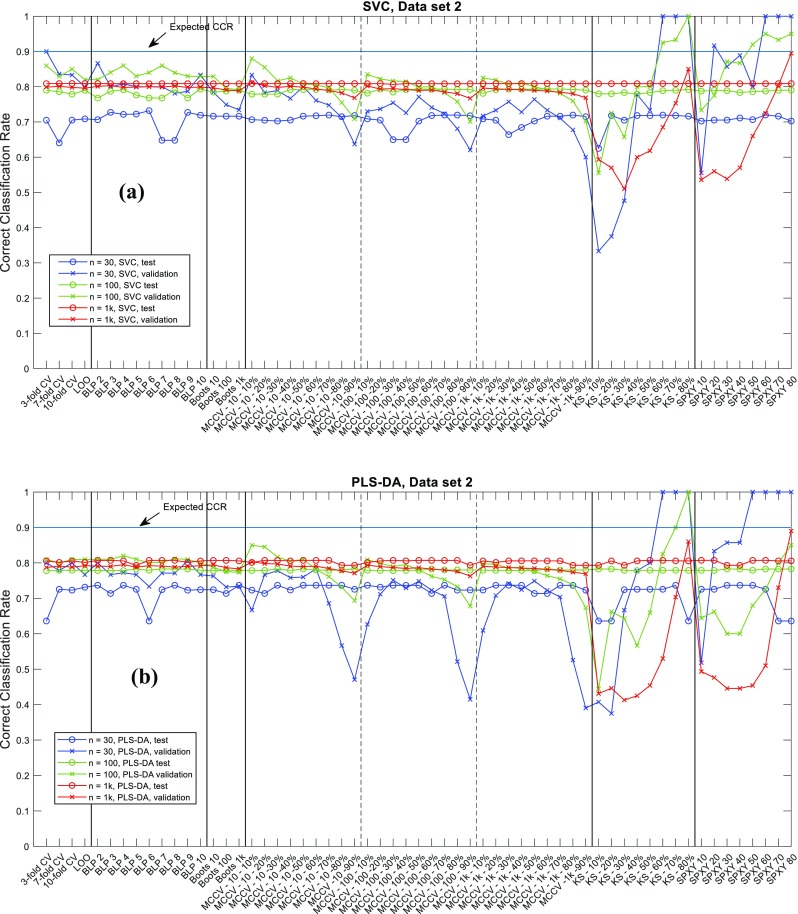
CCRs on data2 (*p*2) for **a** PLS-DA and **b** SVC

**Fig. 6:**
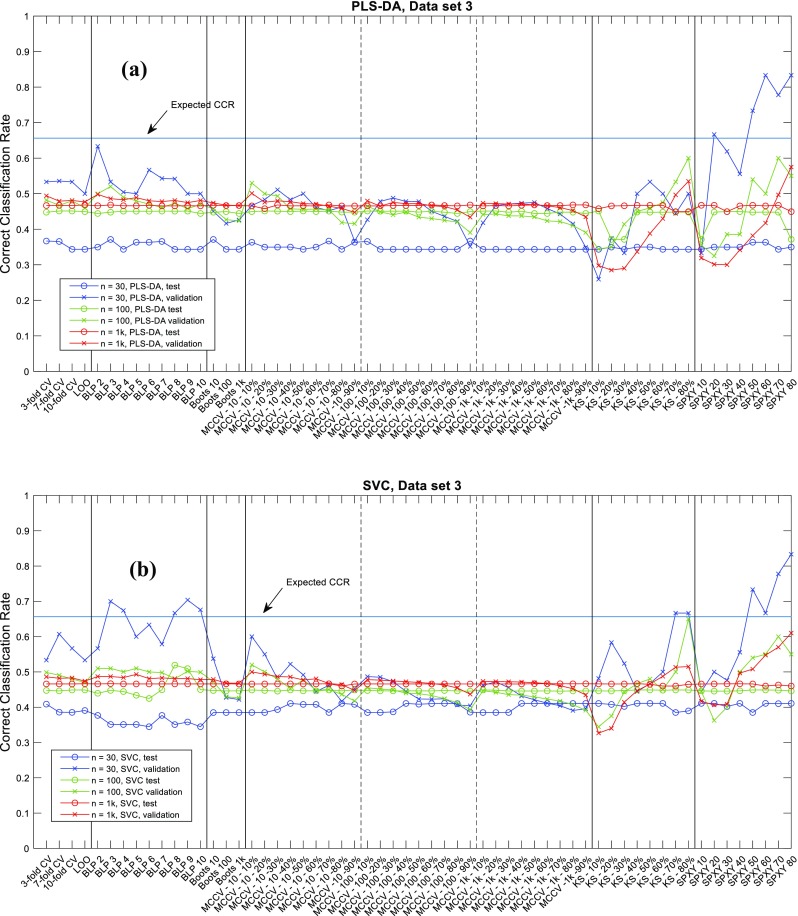
CCRs on data3 (*p*3) for **a** PLS-DA and **b** SVC

The correct classification rate (CCR) of all the simulations are provided in an EXCEL spreadsheet named “results_summary.xlsx” as electronic supplementary material (ESM). Graphical presentation of the CCRs on data1, data2 and data3 are given in Figs.4, 5, 6 respectively. The effect of the dataset size (30, 100 or 1000 samples) is the most obvious influential factor. The variation in CCRs of both validation and test sets reduced significantly when the number of samples increased. With 1000 samples available, the CCRs obtained from using the different data splitting methods had almost become a constant. This suggests that when sufficiently large number samples are available the choice of data splitting method and its parameter become much less important, and that all partitioning methods approximate the different normal distributions of the different classes in these populations; that is to say these models are approaching the central limit theory for the population distributions. However, on small datasets with only 30 samples available, it is evident that the CCRs of validation sets varied very significantly and the low CCRs on test sets was evident. This highlights the need to have an appropriate parameterized data splitting method if one wants to have a best possible model on a small dataset. This is especially important for clinical investigations as most metabolomics studies use very small cohorts in case–control disease classifications [26, 27].

Regardless of sample size, the variations in the CCRs on the validation sets was always larger than those for the blind test data, especially with small datasets with only 30 samples. In general, we found that the CCRs of validation sets were higher than those of test sets, indicating that the CCR of validation set was usually an over-optimistic estimation of the model generalization performance compared to test set; this is consistent with previous findings [2]. The two systematic sampling methods—K-S and SPXY—showed the largest variations in CCRs of validation sets, particularly on two ends of parameter settings: when too few samples (10–20%) were used for training, the estimated CCRs were generally over-pessimistic (i.e., lower than those in test sets); in contrast, when too many samples (> 50%) were selected for training, the estimated CCRs were generally over-optimistic (i.e., higher than those on test sets).

When we inspected CCRs for data2 and data3 several settings of K-S and SPXY had achieved perfect classification (i.e., CCR = 100%), and this was when enough samples had been selected for training. SPXY seemed to generate more over-optimistic estimations than K-S. For example, on data1, containing 30 samples, with the SVC model, when only 30% of the samples were used for training SPXY had achieved 100% CCR on the validation set while for K-S at least 70% samples were needed to achieve the same CCR. When 30–40% samples selected for training, the CCRs on the validation sets were sometimes close to the test set CCR. Despite these observations, the gaps between these two types of CCRs were still much wider than other data splitting methods. This suggested that these systematic sampling methods may be too good at selecting the most representative samples from the model performance estimation point of view, because a representative sample set is also required for a realistic estimation of modelling error. It is worth noting that there are other systematic data splitting methods [28–30] which considered the representative of both training and test set and these methods may perform better. Interestingly, despite poor estimations of model performance on the validation sets, the model parameter selected using these two methods were in fact mostly reasonable and the CCRs on the test sets were similar to the other data splitting methods, except a few extreme cases when too many samples had been used for training and these resulted in 100% CCR. Under such circumstances it was impossible to tell the differences between the different model parameters and this resulted in an overly simplistic model (the in-house model selection script was written in a way that if models with different parameters generated the same best CCR, the script would favor the simplest model). This suggested that model selection itself does not require an accurate estimation of generalization performance of the model, a certain amount of systematic bias can be tolerated, and the small variations in test set also confirmed this. For other splitting methods, although these had less variation in CCRs on validation sets, such variations were still significantly higher than those of test sets. Again, such differences were also most evident when the number of samples was small. Similarly, when there were either too many or too few samples in the training set, the gaps between the two types of CCRs were the widest. Again, this highlighted the need to have a balanced training and validation set to have a reasonable estimation of the predictive performance of the model. Imagine that when someone tries to build a classification model on data with large overlaps, it is intuitive to think that the most convenient way to improve the performance of the model is to increase the size of the training set. However, in real-world scenarios where samples may be hard to obtain, no extra samples are available, this would consequently decrease the size of test set and thus result in an even worse estimation of the model’s performance.

Figures 4, 5, 6 are illustrative summaries of the CCRs for all the models and also show that no data splitting method had any obvious advantage over others in finding the optimal model parameters. Instead, most data splitting methods with many different parameter settings resulted in similar CCRs on the test sets. A summary of the maximum, minimum and median CCR, as well as the improvement of the best CCR over the median CCR are given in Tables 2 and 3. For 15 out of 18 cases the best model provided no more than 3% improvement over the corresponding median CCR. The other three models were exceptions and these were for data3 (*n* = 30) with PLS-DA classification; data1 (*n* = 30) with SVC; and data3 (*n* = 100) also with SVC. However, these three cases were from different data splitting methods: the best model for data3 (*n* = 30) with PLS-DA was found by BLP (with *k* = 3) and bootstrap (*t* = 10); the best model for data1 (*n* = 30) by SVC was found by MCCV (*t* = 100, *n*_*t*_ = 30 × 50% = 15); and the best model for data3 (*n* = 100) with SVC was found by BLP (*k* = 8). This suggests that despite employing a wide range of parameter settings for each data splitting methods, it was rare to find a model parameter that was significantly better than the other data splitting methods (including with different settings) and it was difficult to decide which method/parameter combinations were the best for model selection. A general impression is that employing a random sampling method (e.g., MCCV or bootstrap) with enough number of repeats (*t* ≥ 100) and a reasonable balance between training and test set (50–70% for training) one was likely to get a good model. In addition, BLP also appeared to be a good model selection method and was often able to find good model parameters, but there is no clear evidence on how many data splits would be best.

**Table 2:** Summary of CCRs for PLS-DA

		Best CCR	Median CCR	Worst CCR	Best CCR improvement over the median
data1	*n* = 30	92.52%	91.89%	63.69%	0.69%
	*n* = 100	96.00%	94.86%	94.70%	1.20%
	*n* = 1000	96.38%	96.35%	95.79%	0.03%
data2	*n* = 30	73.68%	72.55%	63.60%	1.56%
	*n *= 100	78.29%	77.92%	77.79%	0.47%
	*n* = 1000	80.72%	80.56%	79.32%	0.20%
data3	*n* = 30	37.13%	34.35%	34.35%	8.09%
	*n* = 100	45.13%	44.82%	37.15%	0.69%
	*n* = 1000	46.84%	46.67%	44.95%	0.36%

**Table 3:** Summary of CCRs of SVC

SVC		Best CCR	Median CCR	Worst CCR	Best CCR improvement over the median
data1	*n* = 30	90.98%	84.44%	83.39%	7.75%
	*n* = 100	95.49%	94.82%	92.14%	0.71%
	*n* = 1000	95.98%	95.94%	94.51%	0.04%
data2	*n* = 30	73.21%	71.49%	62.49%	2.41%
	*n* = 100	79.34%	78.99%	76.79%	0.44%
	*n* = 1000	80.92%	80.87%	80.68%	0.06%
data3	*n* = 30	41.08%	40.21%	34.47%	2.16%
	*n* = 100	51.92%	44.63%	42.47%	16.33%
	*n* = 1000	46.66%	46.59%	46.00%	0.15%

**Fig. 7:**
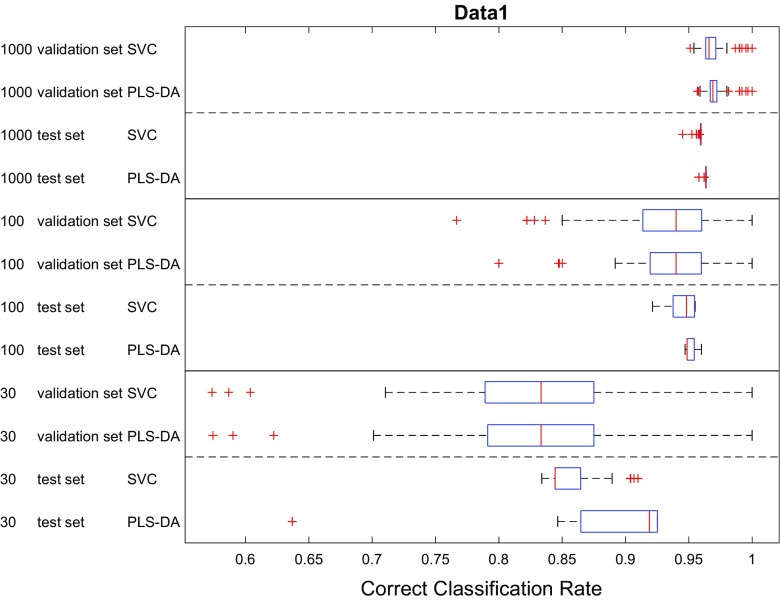
Comparison showing the CCR distributions as box–whisker plots for PLS-DA and SVC analyses on data1 (*p*1). In these box–whiskers the red line is the median CCR, the top and bottom of the boxes are the 25th and 75th percentiles; the size of the box is the interquartile range (IQR); the whiskers extend to the most extreme data points which are not considered as outliers (red crosses), defined as no more than 1.5 × IQR outside of the IQR

**Fig. 8:**
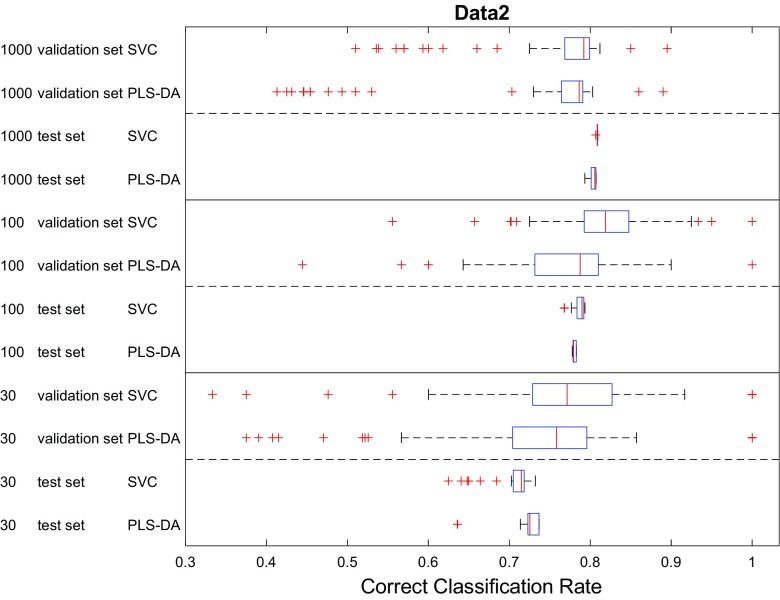
Comparison showing the CCR distributions as box–whisker plots for PLS-DA and SVC analyses on data2 (*p*2)

**Fig. 9:**
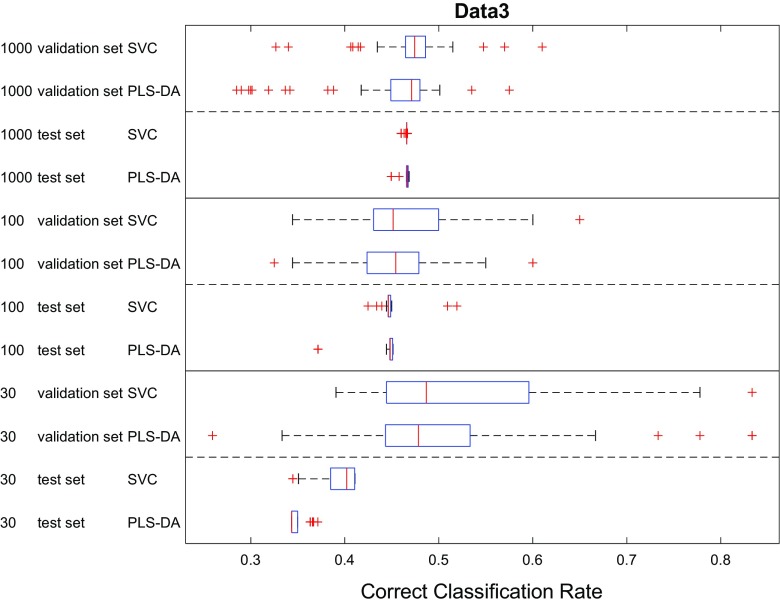
Comparison showing the CCR distributions as box–whisker plots for PLS-DA and SVC analyses on data3 (*p*3)

Finally, we also compared the classification performances of PLS-DA and SVC. The CCRs of these two models using different data splitting methods were pooled and the results presented as box–whisker plots in Figs. 7, 8, 9 for the three datasets. In most cases, the performance of these two models were very close to each other on the test set as seen by the overlaps in the CCR distributions. For the two datasets with smaller overlaps (Fig. 3) and low sample numbers (*n* = 30) PLS-DA performed slightly better than SVC on data1 (*p*1) and data2 (*p*2); by contrast, when the data had more overlap SVC showed significantly better performance on data3 (*p*3 with 30 samples). Moreover, despite the fact that the data were generated from simple multivariate mixed normal distributions in MixSim, where we did not include additional random noise on the input variables and did not include outliers, the best CCRs on the largest datasets provided by these two models were still lower than the expected CCR (Figs. 7, 8, 9) and the differences in CCRs were larger on data with more overlap (viz. data3). On data1 the best CCR of trained model achieved 98.85% of expected CCR, 89.91% on data2 and only 71.62% on data3. This was most likely due to the limitations of classification model itself and suggests that there is still large room for further development of modelling algorithms—although we recognize that if the data contains misclassifications we can not improve on that.

## Conclusion

In this study, we conducted a comprehensive comparison study on various different data splitting methods for model selection and validation. To have predetermined classifications we based this on simulated data using MixSim which we used to generate nine simulated datasets each with 10 input variables based on finite mixed normal distribution with different probabilities of misclassification (Table 1) and variable sample sizes. We chose sample sizes of 30, 100 and 1000 as many studies reported in the metabolomics literature [26] typically have small sample sizes of between 30 and 100.

The results suggested that most methods with typical parameter settings resulted in similar correct classification results (Figs. 4, 5, 6, 7, 8, 9 and see ESM), therefore, they are all viable options for model selection. However, estimating errors on the validation datasets proved to be very sensitive to the choice of data splitting method used to partition the training data into training and validation sets, as well as its parameter setting, especially when small datasets with just 30 samples were used. To have a stable estimation of model performance, a good balance between training and test set is required. Also, there is no clear evidence suggesting which method/parameter combination would always give significantly better results than others. This perhaps expected within the chemometrics arena—There is no free lunch!—therefore, the choices of which method to use for data splitting and which parameters to use cannot be decided a priori and would be data dependent.

The MixSim model was very useful as this allowed us to generate a dataset with a known probability of misclassification. This enabled us to compare the generalization performance estimated from the data against the “true” answers and we found that even the performance of the best model cannot reach the expected/known correct classification rate.

In conclusion, we found that model performance improved when more samples were used and this is in agreement with metabolomics studies where 300 or more subjects per class are recommended to effect good classification [31]. However, as reported [26] this is rarely the case and perhaps why there are so many false discoveries in metabolomics [27] and biomarker discovery in general [32].

## Electronic supplementary material

Below is the link to the electronic supplementary material.

Supplementary material 1 (XLSX 44 kb)
